# Chromosomally normal miscarriage is associated with vaginal dysbiosis and local inflammation

**DOI:** 10.1186/s12916-021-02227-7

**Published:** 2022-01-28

**Authors:** Karen Grewal, Yun S. Lee, Ann Smith, Jan J. Brosens, Tom Bourne, Maya Al-Memar, Samit Kundu, David A. MacIntyre, Phillip R. Bennett

**Affiliations:** 1 Tommy’s National Centre for Miscarriage Research, Institute of Reproductive & Developmental Biology, Imperial College London, Hammersmith Hospital Campus, Du Cane Road, London, UK; 2 March of Dimes, European Preterm Birth Research Centre, Imperial College London, Hammersmith Hospital Campus, Du Cane Road, London, UK; 3 Faculty of Health and Applied Sciences, University West of England, Bristol, UK; 4 Tommy’s National Miscarriage Research Centre, Division of Biomedical Sciences Warwick Medical School, University of Warwick, Coventry, UK

**Keywords:** Miscarriage, Microbiota, Infectious disease, Early pregnancy, Translational research

## Abstract

**Background:**

Emerging evidence supports an association between vaginal microbiota composition and risk of miscarriage; however, the underlying mechanisms are poorly understood. We aim to investigate the vaginal microbial composition and the local immune response in chromosomally normal and abnormal miscarriages and compare this to uncomplicated pregnancies delivering at term.

**Methods:**

We used 16S rRNA gene based metataxonomics to interrogate the vaginal microbiota in a cohort of 167 women, 93 miscarriages (54 euploid and 39 aneuploid using molecular cytogenetics) and 74 women who delivered at term and correlate this with the aneuploidy status of the miscarriages. We also measured the concentrations of IL-2, IL-4, IL-6, IL-8, TNF-α, IFN-γ, IL-1β, IL-18 and IL-10 in cervical vaginal fluid.

**Results:**

We show that euploid miscarriage is associated with a significantly higher prevalence of *Lactobacillus* spp*.* deplete vaginal microbial communities compared to aneuploid miscarriage (*P* = 0.01). Integration of matched cervicovaginal fluid immune-profiles showed that *Lactobacillus* spp*.* depleted vaginal microbiota associated with pro-inflammatory cytokine levels most strongly in euploid miscarriage compared to viable term pregnancy (IL-1β; *P* < 0.001, IL-8; *P* = 0.01, IL-6; *P* < 0.001).

**Conclusions:**

Our data suggest the vaginal microbiota plays an important aetiological role in euploid miscarriage and may represent a target to modify risk of pregnancy loss.

**Supplementary Information:**

The online version contains supplementary material available at 10.1186/s12916-021-02227-7.

## Background

Miscarriage, defined as pregnancy loss before the fetus reaches viability, is a distressing disorder associated with pain and bleeding as well as significant psychological morbidity. Early miscarriage (pregnancy loss before 12 weeks) occurs in one in five pregnancies of which half are due to chromosomal abnormalities [1]. Infection is implicated in 66% of late miscarriages (12–24 weeks) but is less prevalent in early miscarriage [2]. However, the mechanisms driving miscarriage in these groups are poorly defined. Despite its prevalence, there are no interventions that prevent sporadic miscarriage and only few treatments, such as progesterone supplements, have been shown to modestly reduce the recurrence risk of miscarriage in subsequent pregnancies [3].

Pregnancy has a unique and dynamic immunological milieu that is required to support a healthy pregnancy [4]. Initially, a pro-inflammatory state is required for implantation which involves a release of inflammatory mediators inducing tissue injury and repair [5]. Infection may disrupt the immunological synergy at implantation and trigger adverse outcome [6]. For example, chlamydial infection has been shown to cause dysregulation of decidualisation [7], which can contribute to miscarriage by impairing implantation and trophoblast invasion. As pregnancy progresses, there is broadly resolution of inflammation until close to term, a pro-inflammatory state then returns and contributes to the mechanisms of the onset of labour [8]. However, a continuous anti-inflammatory state in pregnancy is considered over simplified and has since been disputed. Further work has described localized changes in response to infection or ‘dangerous’ signals [9]. Therefore, failed tolerance in certain women at the maternal fetal interface and inappropriate, premature activation of inflammation in the reproductive tract may lead to miscarriage or preterm birth [10].

Emerging evidence implicates the reproductive tract microbiota as a key modulator of local inflammatory and immune pathways throughout pregnancy. During pregnancy, increased oestrogen levels promote glycogen deposition in the vaginal epithelia, consequently supporting *Lactobacillus* spp. dominance and stability during pregnancy [11–13]. This relationship provides protection against pathogenic bacteria by producing lactic acid and antimicrobial compounds such as bacteriocins [14]. Depletion in vaginal *Lactobacillus* spp. is linked to adverse pregnancy outcomes, including preterm birth and preterm prelabour rupture of fetal membranes (PPROM) [15–17]. There is a correlation between vaginal communities deplete in *Lactobacillus* spp. and levels of pro-inflammatory cytokines within the cervicovaginal fluid (CVF), suggesting a mechanistic link between an aberrant vaginal microbial environment and adverse pregnancy outcome [18, 19].

In a recent longitudinal study of the pregnancy vaginal microbiome starting at 6 weeks of gestation, we showed that women with vaginal communities dominated by species other than *Lactobacillus* spp. at any point during gestation were at increased risk of PPROM [20]. We have also recently shown that a *Lactobacillus* spp. depleted vaginal microbiome in early pregnancy is a risk factor for first trimester miscarriage [21]. However, this study did not distinguish between euploid and aneuploid pregnancy losses, nor did it explore the relationships between the vaginal microbiota and inflammatory mediators. We hypothesise that whilst aneuploid pregnancies are lost mainly because of intrinsic developmental errors, euploid miscarriages may be caused by inflammatory signals triggered by an adverse vaginal microbiota composition. To test this hypothesis, we characterized the vaginal microbiota and host immune response in women with chromosomally normal and abnormal miscarriages as well as in uncomplicated pregnancies that successfully progressed to term.

## Methods

### Study population and study design

This study was a prospective observational cohort study based at Queen Charlotte’s & Chelsea Hospital, Early Pregnancy Unit, London between March 2014 and February 2019. The study was approved by NHS National Research Ethics Service (NRES) (REC 16/WA/0357 and REC 14/LO/0199). All participants provided written informed consent. Patients were not involved in the development of the research. Patients were recruited either when they presented initially with a confirmed miscarriage diagnosis, or when they presented with pain and/or bleeding without an initially confirmed miscarriage diagnosis in the first trimester of pregnancy. The first trimester was defined as < 14 weeks’ gestation by last menstrual period (LMP) or, where LMP was not known, ultrasound scan dating based on crown-rump length measurements (CRL). An intrauterine pregnancy was defined on the basis of an ultrasound scan showing an intrauterine gestation sac with or without a visible embryo and heartbeat. Missed miscarriage was confirmed when an empty gestation sac was present with a mean sac diameter of 25 mm or more, if an embryo with CRL measurement of 7 mm or more was identified without an embryonic heartbeat or if the embryonic heartbeat was absent irrespective of the size of the CRL, if one had previously been observed [22, 23]. A diagnosis of incomplete miscarriage was made when a transvaginal ultrasound demonstrated irregular heterogeneous tissue in the endometrial cavity in keeping with retained products of conception after a previous ultrasound scan had shown an intrauterine pregnancy [24].

Participants were recruited via open advertisements (using posters) within the hospital and at the university where the study was being conducted (Imperial College). The majority of women were recruited after attending the hospital Ultrasound Department or Early Pregnancy Assessment Unit. Exclusion criteria for this study included women under 18 years of age, sexual intercourse within 72 h of sampling and human immunodeficiency virus (HIV) or hepatitis C-positive status. All patients using antibiotics, probiotic supplements or progesterone supplements within 2 weeks of sample collection were excluded. A detailed questionnaire including demographic information, past medical, gynaecological and obstetric history was completed. Validated symptom scores were used to assess vaginal bleeding based upon a pictorial blood assessment chart score at the time of sampling [25]. In this methods, bleeding score 0 represents no bleeding, 1 minimal bleeding, 2 moderate bleeding, 3 soaking sanitary towels and 4 passing clots.

### Sample collection

Cervicovaginal fluid samples were collected from each participant from the posterior vaginal fornix using a BBL CultureSwab MaxV Liquid Amies swab (Becton, Dickinson and Company, Oxford, UK) prior to surgical management of miscarriage (the minimum time between transvaginal ultrasound scan and sample collection was 48 h). Swabs were stored in 500 μl of liquid amies and immediately placed on ice before being frozen and stored at − 80 °C within 5 min of collection. A subset of swabs (*n* = 96) were directly stored at − 80 °C without liquid amies. All swabs were weighed before and after collection to determine the wet weight. Negative control swabs were also collected by exposing swabs to clinic and laboratory environments prior to freeze storage. The degree of vaginal bleeding was assessed at the time of surgical evacuation.

### Cytogenetic analysis

Chorionic villous material was collected at the time of surgical evacuation of the uterus and analysed for molecular cytogenetics using QF-PCR (quantitative fluorescent polymerase chain reaction) or BACs (bacterial artificial chromosomes) on Beads [26]. For molecular cytogenetics using QF-PCR, DNA was amplified using two multiplexes that include a total of 31 markers; assay 1 contains primers for chromosomes 13, 18, 21 and 22, and assay 2, primers for chromosomes 14, 15 and 16 and the X and Y chromosomes. Supplementary markers were used as required. PCR products were separated on an ABI 3100 capillary genetic analyser, and results were analysed using ABI Genotyper software [27]. The KaryoLite bacterial artificial chromosomes-on-Beads (KL-BoBs™) assay was performed using a prenatal chromosome aneuploidy and microdeletion detection test kit (Perkin Elmer, Waltham, MA, USA), according to the manufacturer’s instructions. Briefly, genomic DNA from specimens as well as reference DNA were biotinylated and purified. The genomic DNA and BoBs™ was then subjected to single-cell hybridization overnight before washing and incubation with streptavidin-phycoerythrin, which was used as the reporter. Fluorescence of DNA bound to the microbeads was measured using a Luminex 200 (Austin, TX, USA), and BoBsoft™ analytical software (Perkin Elmer) was used for data analysis whereby a ratio of specimen fluorescence to reference fluorescence greater than 1.0 indicated the chromosome fragments were repeated and a ratio less than 1.0 indicated a deletion [26].

### DNA extraction and bacterial 16S rRNA gene amplicon sequencing

The methods for DNA extraction from the vaginal swabs (BBL^TM^ CultureSwab^TM^) were followed from those outlined in the Manual of Procedures for the Human Microbiome Project with minor modifications [28]. The details of the DNA extraction from vaginal swabs was performed as previously outlined [12]. In brief, mixed universal primers 28F-YM GAGTTTGATYMTGGCTCAG, 28F-Borrellia GAGTTTGATCCTGGCTTAG, 28F-Chloroflex GAATTTGATCTTGGTTCAG and 28F-Bifdo GGGTTCGATTCTGGCTCAG at a ratio of 4:1:1:1 with 388R TGCTGCCTCCCGTAGGAGT reverse primers were used to amplify the V1-V2 region of 16S rRNA. Sequencing was performed at RTL genomics (Lubbock, TX, USA) using the Illumina MiSeq platform (Illumina Inc.). The data was processed and analysed using the MiSeq SOP Pipeline of the Mothur package [29]. Sequence alignment was performed using blastn (16SMicrobial.tar.gz) and classification used RDP (Ribosomal Database Project) [30]. To account for sequencing depth bias, data were resampled and normalised to the lowest read count. Rarefaction curves were constructed and analysed to guide selection of depth for rarefaction and Good's coverage index calculated to facilitate assessment of adequacy of sub-sampling of the data.

### Cytokine analysis

The vaginal swabs used for microbiome analysis were thawed slowly on ice and vortexed. For the subset of swabs not stored in amies solution, a total of 500 μl of PBS supplemented with 2.5 μl protease inhibitor (PI) was added to the swab to provide a comparable dilution volume as those stored in Amies transport solution. A constant volume was used as the mean wet weight for all swabs were highly comparable (mean 0.07 g ± 0.02). The samples were then centrifuged (8000 rpm for 10 min) and the supernatant removed and stored at − 20 °C for cytokine studies. In cases where supernatant was not collected and only dry swabs were available, we suspended the swab in protease inhibitor (PI) and PBS (5 μl PI/1 ml PBS) to make up the same volume of as those stored in Amies transport solution. The supernatants (50 μl) were analysed by Human Magnetic Luminex Screening Assay (8-plex) to measure the concentration of the following analytes: IL-2, IL-4, IL-6, IL-8, TNF-α, IFN-γ, IL-1β, IL-18 and IL-10.

### Statistical analysis

Analysis of statistical differences between the vaginal microbiota of samples according to pregnancy outcome was performed using hierarchical clustering analysis (HCA) using Ward linkage in CLUSTVIS (https://biit.cs.ut.ee/clustvis). Vaginal microbiota composition was classified into two groups at genus level according to the relative abundance of *Lactobacillus* spp. using Ward hierarachial clustering analysis; samples were also classified into CSTs using the recently developed *VA*gina*L* community state typ*E N*earest *C*entro*I*d classifier (VALENCIA) [31]. The Fisher’s exact test was used when comparing *Lactobacillus* spp. depleted and dominated in three different pregnancy outcome categories.

Linear discriminant analysis (LDA) effect size (LEfSe) analysis was used to identify taxa significantly overrepresented according to clinical outcome, through all taxonomic levels [32]. This analysis was performed using taxonomic relative abundance, with per-sample normalisation and default settings for alpha values (0.05) for the factorial Kruskal–Wallis test among classes and pairwise Wilcoxon test between subclasses. A logarithmic LDA score > 2 was used to determine discriminative features.

Other statistical analyses were performed using the statistical package GraphPad Prism v.8.4.3 (GraphPad Software Inc., CA, USA).

To infer the pattern of microbial relationships in the vaginal microbiome, we used BAnOCC [33] to construct correlation matrices at the genus and species level (we limited the latter to the top 50 species with the greatest overall abundance across the entire cohort). The MCMCs were run for 10,000 iterations and four chains (runs were checked for convergence). Co-occurrence networks were drawn using these correlation matrices in the Qgraph package in R [34].

## Results

### Patient cohort, characteristics and outcomes

**Fig. 1:**
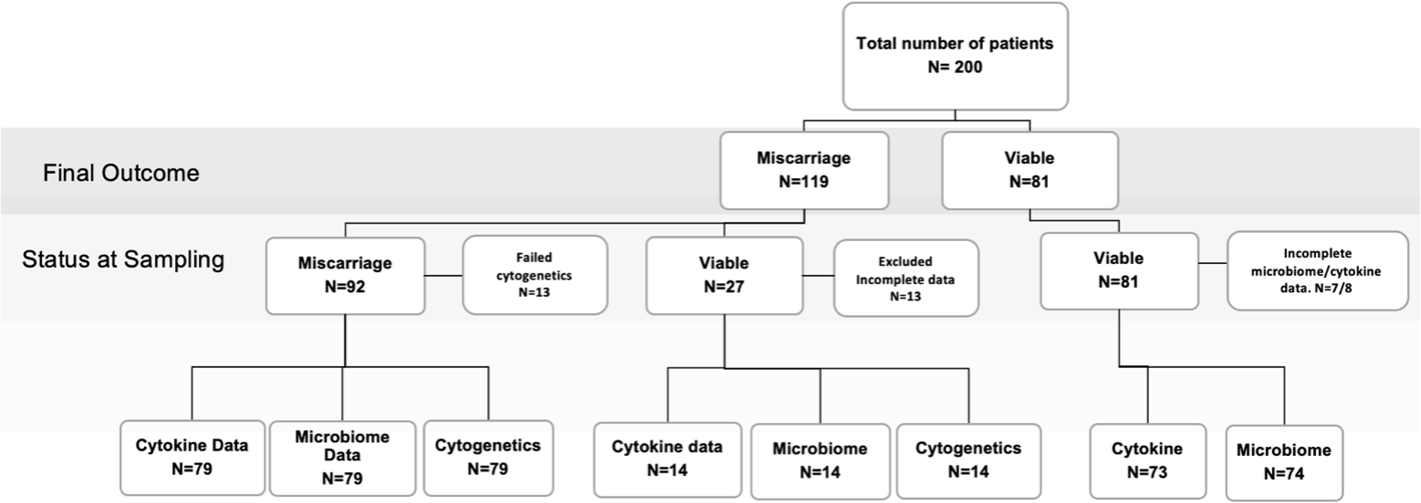
Flowchart detailing entire study cohort including status at sampling and experiments performed

Two hundred women were recruited, of whom 119 miscarried and 81 had a viable term pregnancy. Of the women who miscarried, 92 were recruited at first consultation, the time of miscarriage diagnosis. A live pregnancy was initially diagnosed in 108 cases of whom 27 went on to miscarry and 81 had a successful pregnancy and delivered at term. Cytogenetic analysis was unavailable in 26 cases (in 24 cases due to inadequate numbers of villi and in two cases because of technical failure). Of the 81 viable pregnancies, eight swabs were unavailable for cytokine analysis and seven unavailable for microbiota analysis. The final study cohort consisted of two patient groups, one miscarriage group for which vaginal microbiota, cytogenetic and vaginal cytokine concentrations were available for all cases and one term pregnancy group for which 74 microbiota data and 73 cytokine data were available. The final cohort therefore comprised a total 93 pregnancies that miscarried and 74 pregnancies that went to term (Fig. 1). Table 1 shows the clinical and demographic characteristics of the three patient groups. Median maternal age was 32 (range 17–46) in the euploid miscarriage group, 36 (range 27–45) in the aneuploid miscarriage group and 32 (range 20–44) in the term pregnancy group. Maternal age was significantly higher in the aneuploid miscarriage group (*P* = 0.0044, Kruskal-Wallis test). Women in the term pregnancy group were significantly less likely to have had one or more previous miscarriages (*P* = 0.0084, chi-squared test). Gestational age at sampling was categorised as 5–8, 8–10, or 10–14 weeks post last menstrual period (LMP). There were no significant differences in gestational age at the time of sampling between aneuploid and euploid miscarriage group, but the term pregnancy group were sampled significantly later (*P* = 0.0083, chi-squared test). Bleeding scores were similar between euploid and aneuploid miscarriage groups and higher bleeding scores were more common in the miscarriage groups compared to the term pregnancy group (*P* = 0.0002, chi-squared test). There were no significant differences in BMI, smoking status or ethnicity between the groups.

**Table 1:** Clinical and demographic characteristics of 167 patients included in study cohort

	Euploid miscarriage	Aneuploid miscarriage	Control (term pregnancy)	*p* value
Number of women	54	39	74	
Maternal age, years Median (range)	32 (17–46)	36 (27–45)	32 (20–44)	^†^0.0044
BMI (kg/m^2^) Median (range)	25.1 (17.7–35)	24.4 (18.7–39.4)	24.5 (18–38.4)	^†^0.205
Smokers (%)	7 (13)	2 (5.1)	3 (3.7)	^††^0.189
Ethnicity (%)				
White	30 (56)	24 (62)	54 (73)	^†††^0.1271
Asian	15 (28)	11 (28)	10 (13.5)	
Black	7 (13)	4 (10)	10 (13.5)	
Mixed	2 (3)	0 (0)	0 (0)	
Previous miscarriage (%)				
0	30(55.6)	22 (56.4)	31 (42)	^††††^0.0084
1	9(16.7)	6 (15.3)	29 (39)	
2	9 (16.7)	7 (18)	10 (14)	
≥ 3	6 (11)	4 (10.3)	4 (5)	
Gestational age group at sampling (%)				
5–8 weeks	14 (26)	14 (36)	7 (10)	^†††^0.0083
8–10 weeks	24 (44)	19 (49)	41(55)	
10–14 weeks	16 (30)	6 (15)	26 (35)	
Bleeding score				
0	28	24	65	^†††^0.0002
1	13	11	9	
2	8	2	0	
3	3	2	0	
4	2	0	0	
Status at sampling				
Missed	40	35		^†††^0.1
Incomplete	4	0		
Viable	10	4		

^†^Kruskal-Wallis test

^††^Fisher’s exact (two-tailed)

^†††^Chi-squared

^††††^Chi-squared combining euploid and aneuploid miscarriages and comparing with viable term pregnancies

### Baseline vaginal microbiota composition and pregnancy outcomes

In total 4,523,582 sequence reads were obtained from 167 samples with an average of 27,087 reads per sample and a median read length of 370 bp after bar code removal. Following removal of singletons and rare operational taxonomic units (OTUs), a total of 128 taxa were identified in the vaginal microbiota of the study cohort. All negative control samples failed to amplify or generate any read data following sequencing apart from 1, which had a read count of 15. By comparison, the average read depth for patient samples was 27,087 (minimum 1402) reads. The sequencing depth was similar across the three groups with an average read count of 25,926 reads for Euploid miscarriage, 25,257 for Aneuploid and 28,899 for Viable ( *p* = 0.1196, Kruskall and Wallis test). To avoid sequencing bias, OTUs were randomly sub-sampled to the lowest read count of 1402 which maintained a minimum Good’s coverage value of 95.9% (range 95.9–99.9%) for all samples. Further analysis was restricted to the top 50 taxa which accounted for 98% of the total sequence reads in the dataset.

**Fig. 2:**
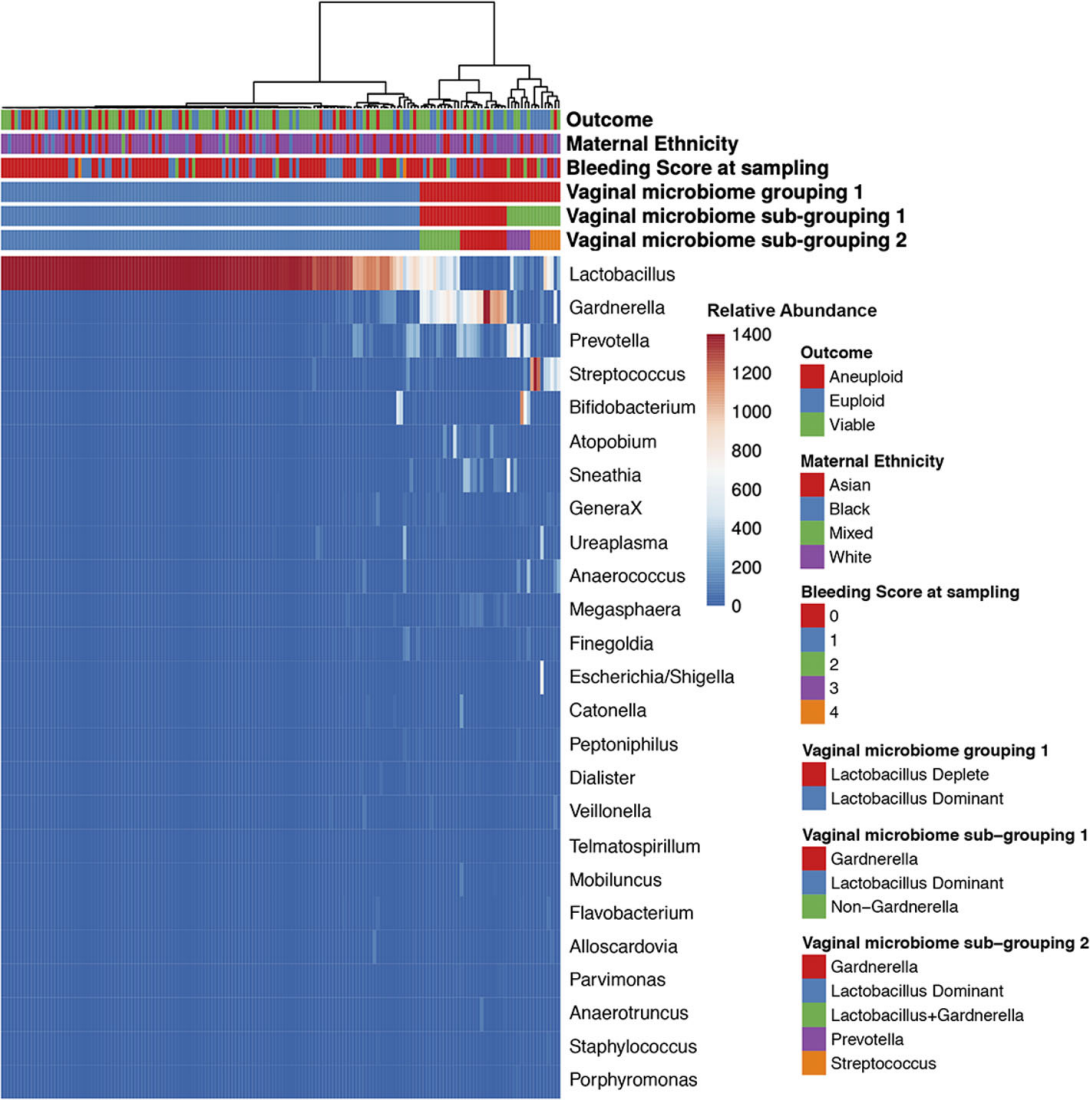
Heat map of relative abundance data for the top 50 most prevalent vaginal bacterial genera and relationship with different clinical outcomes. Mixed universal primers 28F-YM GAGTTTGATYMTGGCTCAG, 28F-Borrellia GAGTTTGATCCTGGCTTAG, 28F-Chloroflex GAATTTGATCTTGGTTCAG and 28F-Bifdo GGGTTCGATTCTGGCTCAG at a ratio of 4:1:1:1 with 388R reverse primers were used to amplify the V1-V2 region of 16S rRNA followed by sequencing using the Illumina MiSeq platform. The data was processed using the MiSeq SOP Pipeline of the Mothur package and classification used RDP. Ward hierarchical clustering of genus level relative abundance data for the whole cohort of 167 individual samples identified two major vaginal microbiome groups (VMG), characterized by *Lactobacillus* spp. dominance 75% (125/167) and *Lactobacillus* spp. depletion 25% (42/167). The mean *Lactobacillus* content in the *Lactobacillus* spp. dominated group was 94.2% and in the *Lactobacillus* spp. deplete group was 18.5%. The *Lactobacillus* spp. deplete cluster was further divided into Gardnerella dominant and non-Gardnerella (VMG sub-grouping 1), and these clusters could be further divided into Gardnerella dominant, mixed Lactobacillus/Gardnerella, Prevotella or Streptococcus dominated (VMG sub-grouping 2)

Ward hierarchical clustering of genus level relative abundance data for the whole cohort identified two major vaginal microbiome groups (VMG), which were characterized by *Lactobacillus* spp. dominated or *Lactobacillus* spp. depleted (vaginal microbiome grouping 1; Fig. 2). These were observed in 75% (125/167) and 25% (42/167) of samples respectively. The *Lactobacillus* spp. dominated group had a mean *Lactobacillus* content of 94.2%. The *Lactobacillus* spp. depleted group had a mean *Lactobacillus* content of 18.5%. The *Lactobacillus* spp. deplete cluster was further divided into Gardnerella dominated and non-Gardnerella (vaginal microbiome sub-grouping 1), and these clusters could be further divided into Gardnerella dominant, mixed Lactobacillus/Gardnerella, Prevotella or Streptococcus dominant (vaginal microbiome sub-grouping 2). Similar analyses were performed at species level to identify the principal *Lactobacillus* spp. present in the *Lactobacillus* spp. dominant group of each individual patient. Ward hierarchical clustering separated patient samples into clusters that were dominated by either *Lactobacillus crispatus* (37%), *Lactobacillus iners* (19%), *Lactobacillus gasseri* (11%), *Lactobacillus jensenii* (10%), *Lactobacillus acidophilus* (2%), *Gardnerella vaginalis* (15%) and a highly diverse group (8%), Additional file 1: Figure S1. Using this classification, no particular *Lactobacillus* spp. was especially protective (chi-squared, *P* = 0.3). Classification of samples into equivalent CSTs using the recently developed VALENCIA classification tool [31] indicated broad agreement between the two clustering approaches (84%) (Additional file 1: Figure S1). In the remaining samples where classification differed, low similarity scores indicated poor fitting to the CSTs pre-defined by the VALENCIA algorithm. This indicated underlying composition differences in samples from our patient cohort and those used to train VALENCIA. The remaining analyses were therefore performed using our defined community clusters.

**Fig. 3:**
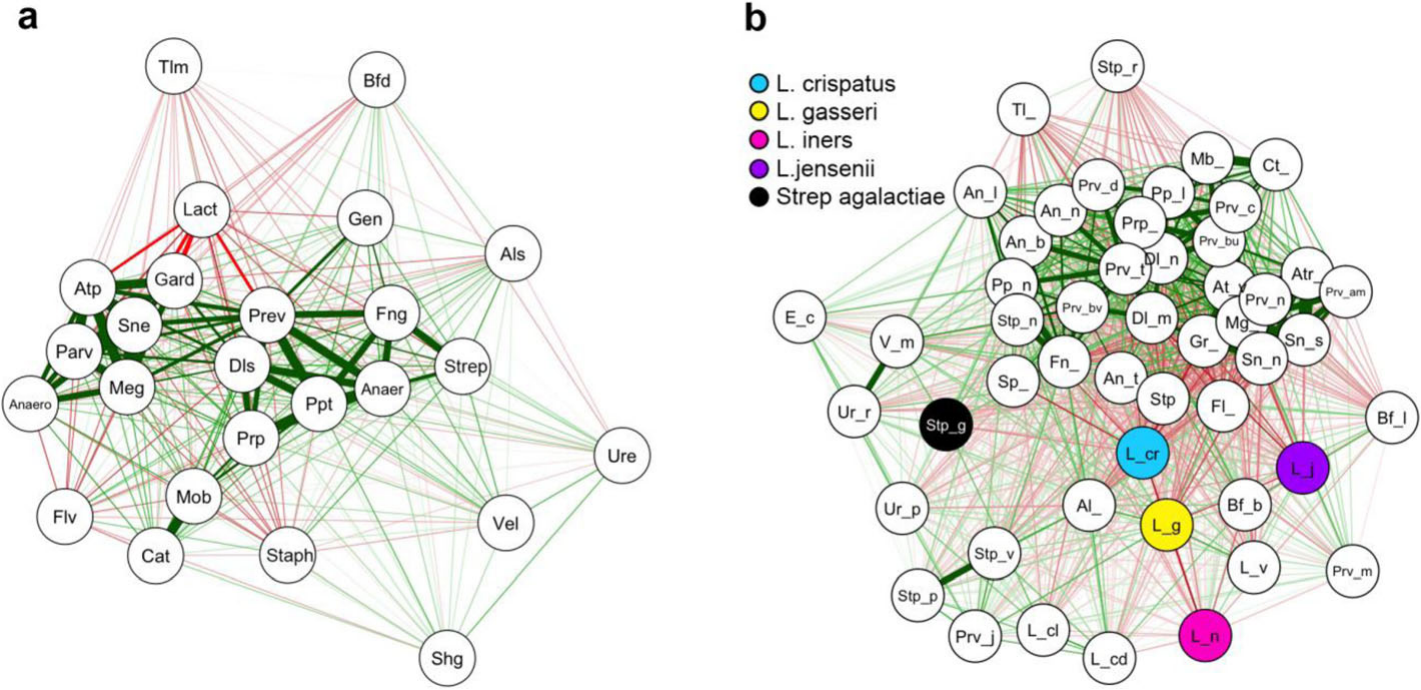
Co-occurrence network analysis of vaginal bacterial taxa in the overall patient cohort. Bayesian Analysis of Compositional Covariance (BAnOCC) was used to generate co-occurrence networks between vaginal microbiota at genera (**a**) and species level taxonomy (**b**) using the 50 species with the highest overall abundance. This programme uses a Bayesian framework to analyse compositional covariance. We ran the MCMC for 10000 generations and four chains and restricted subsequent analyses to edges with [*r*] > 0.3. The red lines indicate negative correlations and the green lines indicate positive correlations and the lines are weighted according to the magnitude of the correlation. There were high levels of co-occurrence between BV associated genera, whereas *Lactobacillus* was negatively correlated with most other genera (**a**). At species level, *L.crispatus* had the most number of negative edges with all other bacterial taxa (**b**)

Analysis of co-occurrence between bacterial genera within our dataset identified a strong relationship between bacterial vaginosis (BV) associated genera including Gardnerella, Atopobium and Prevotella (Fig. 3a). In contrast, *Lactobacillus* was negatively correlated with most other genera, especially Gardnerella, Sneathia, Atopobium and Megasphaera. When analysis was undertaken at the species level BV-associated taxa were again observed to positively correlate whereas *Lactobacillus* species, especially *L. crispatus*, tended to be negatively correlated with other bacterial taxa, including with other Lactobacilli. Strong co-occurrence between *Streptococcus vestibularis* and *Streptococcus pneumoniae* was also observed and *Streptococcus urinalis* showed a high density of negative edges with BV-associated bacteria but a positive edge with *Streptococcus agalactiae* (Fig. 3b).

**Fig. 4:**
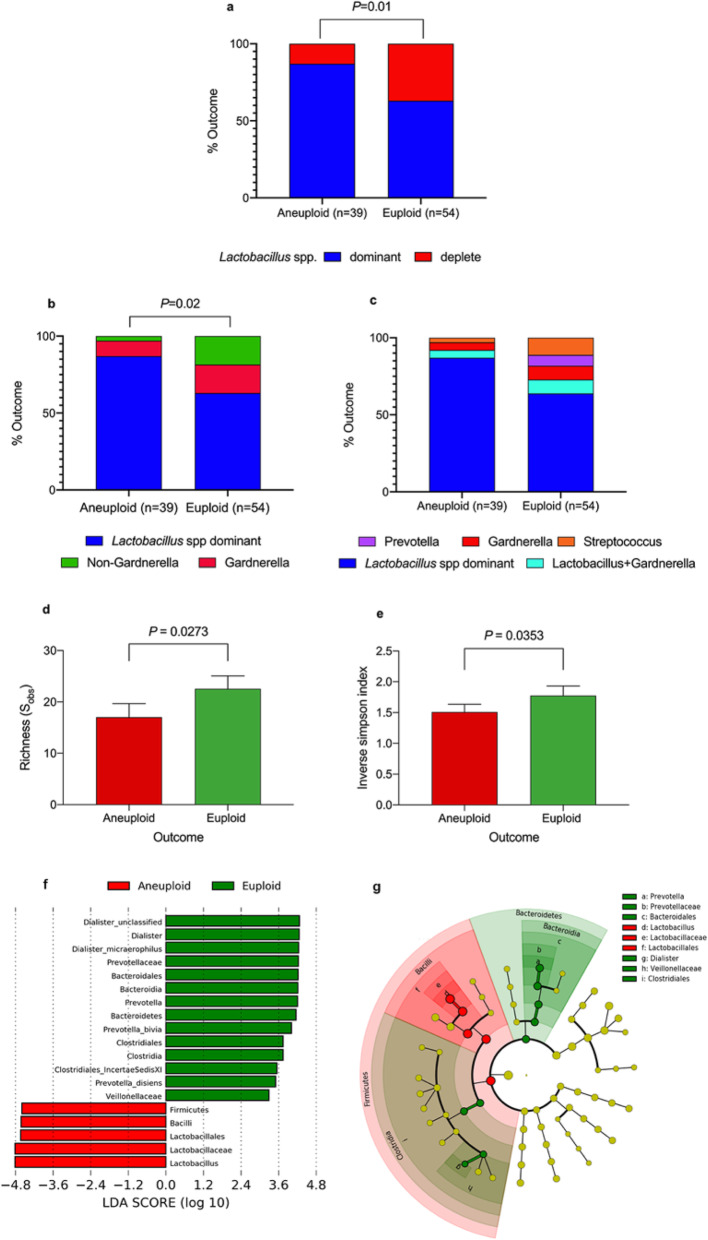
Miscarriage outcome according to vaginal microbial classification. Stacked bar chart illustrating increased representation of *Lactobacillus spp.* depleted vaginal microbial communities in euploid miscarriages compared to aneuploid miscarriages (*P* = 0.01, two tailed Fisher’s exact test **a**). Analysis of vaginal microbiome subgroups showed that this difference was largely driven by non-Gardnerella-dominance of the vaginal niche (*P* = 0.02, two-tailed Fisher’s exact test **b**) and increased relative abundance of *Streptococcus* and *Prevotella* species in euploid miscarriages compared to aneuploid miscarriages (**c**). Consistent with this, significantly higher richness (*P* = 0.0273, two-tailed Mann-Whitney *U*, **d**) and diversity (*P* = 0.0353, two-tailed Mann-Whitney *U*, **e**) was observed in euploid miscarriage compared to aneuploid miscarriage. The linear discriminant analysis (LDA) effect size (LEFse) method (**f**) was then used to identify differentially abundant taxa in euploid compared to aneuploid miscarriage, which were presented at differing taxonomic levels using a cladogram (**g**). Data represented as percentages a, b and c and mean ± standard error of mean in **d** and **e**. LEfSe analysis (**f**, **g**) depicting particular vaginal microbial taxa associated with different clinical outcomes

We next compared vaginal microbiota composition between women who miscarried according to the pregnancy karyotype. Euploid miscarriage was associated with a significantly higher proportion of *Lactobacillus* spp. deplete VMG when compared to aneuploid miscarriage (*P* = 0.01, two-tailed Fisher’s exact test; Fig. 4a). This difference remained significant when correcting for bleeding score by removing patients with a bleeding score > 1 (*P* = 0.02, two-tailed Fisher’s exact test; data not shown). Euploid miscarriage was characterised by a significantly higher proportion of non-Gardnerella *Lactobacillus* spp. depleted VMG (*P* = 0.02, two-tailed Fisher’s exact test, Figure 4b) which was enriched for *Streptococcus* spp. in 60% of cases and *Prevotella spp.* in 40% of cases (Fig. 4c). Consistent with these findings, both bacterial richness and alpha diversity were higher in the euploid miscarriage group (Fig. 4d, e). LEfSe analysis similarly identified decreased levels of *Lactobacillus* and increased levels of *Prevotella*, *Bacteriodia*, *Clostridia* and *Dialister* as characteristic features of euploid miscarriage compared to aneuploid miscarriage (Fig. 4f, g).

**Fig. 5:**
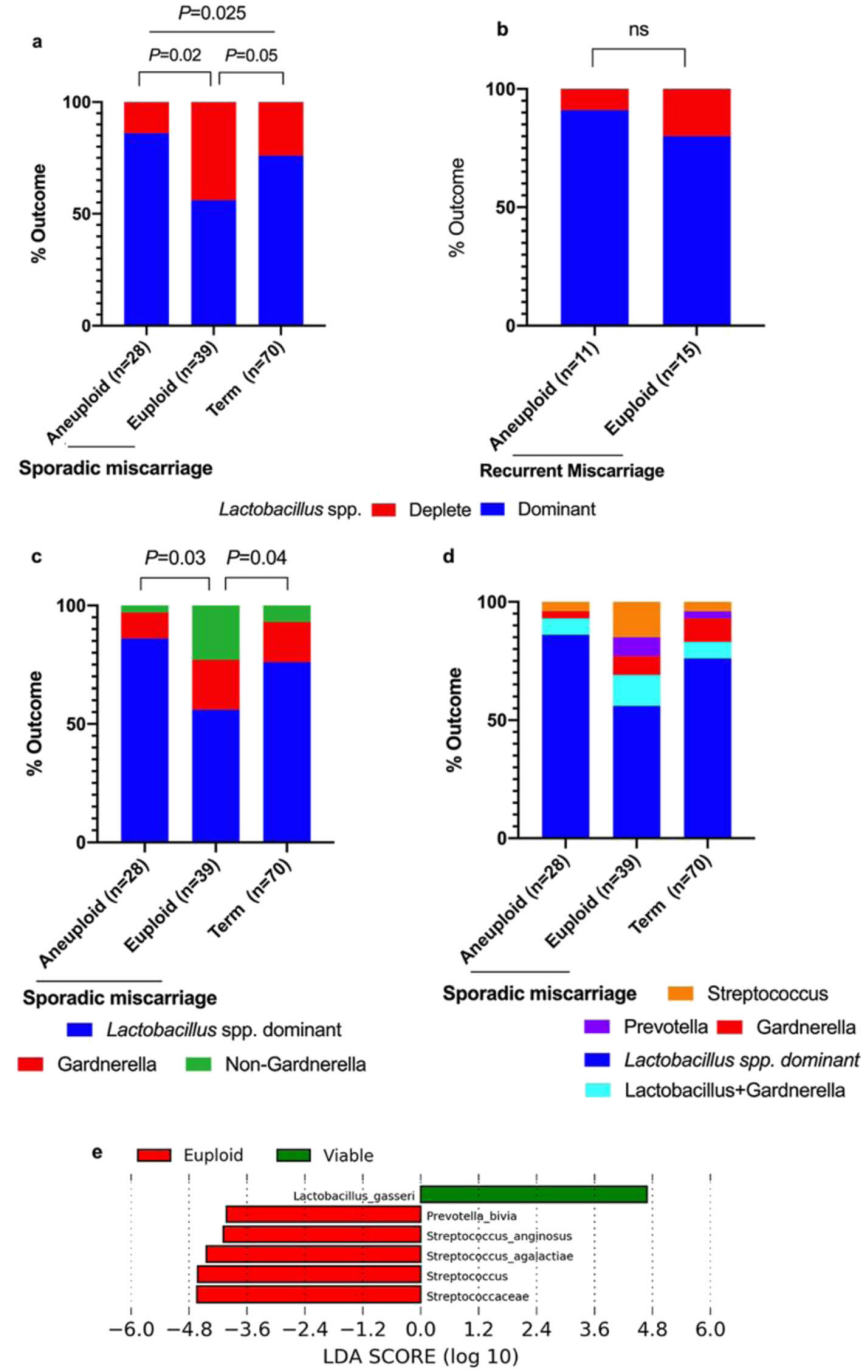
Clinical outcomes according to vaginal microbial composition in sporadic miscarriage. Increased *Lactobacillus spp.* depleted vaginal microbial communities were observed in sporadic euploid miscarriages (*n* = 39) compared to sporadic aneuploid miscarriages (*n* = 28, *P* = 0.02 two-tailed Fisher’s exact test) and viable term pregnancies (*n* = 70 *P* = 0.05 two-tailed Fisher’s exact test, **a**). No significant difference was seen between euploid (*n* = 15) and aneuploid (*n* = 11) miscarriages in the recurrent miscarriage group (**b**). A significantly increased prevalence of non-Gardnerella vaginal bacterial communities was seen in euploid miscarriages compared to aneuploid miscarriage, *P* = 0.03 and viable pregnancy *P* = 0.04 (two-tailed Fisher’s exact test, **c**). Differentially abundant taxa identified by LDA in euploid miscarriage compared to viable pregnancy are shown in **e**. Data represented as percentages in **a**, **b**, **c** and **d**. LEfSe analysis (**e**) depicting particular vaginal bacterial taxa associated with euploid and viable pregnancy, confined to sporadic miscarriage

In our cohort, 26 women had at least two previous miscarriages, prior to a further miscarriage in the index pregnancy and were therefore defined as having ‘recurrent miscarriage’ [35]. Within these cases, there was no difference in the proportion of *Lactobacillus* spp. deplete VMG or *Lactobacillus* spp. dominant VMG between euploid and aneuploid miscarriage (Fig. 5b). Within the sporadic miscarriage and viable term pregnancy group the proportion of *Lactobacillus* spp. deplete VMG was greater in the euploid miscarriage group than either aneuploid miscarriage or viable pregnancy groups (*P* = 0.02 and *P* = 0.05 respectively, two-tailed Fisher’s exact test, Fig. 5a). The prevalence of non-Gardnerella *Lactobacillus* spp. depleted VMG was also greater in the euploid miscarriage group than either aneuploid miscarriage or viable pregnancy groups (*P* = 0.03 and *P* = 0.04 respectively, two-tailed Fisher’s exact test, Fig. 5c) and was particularly associated with *Streptococcus* spp. dominated compositions (Fig. 5d). Although the prevalence of *Lactobacillus* spp. depleted VMG was greater in the euploid miscarriage than the viable pregnancy groups, richness and diversity were not significantly different between the two groups. Similar results were obtained when re-analysis was performed on only those women where recurrent miscarriage was defined as being three consecutive miscarriages with no live births (*n* = 13, Additional file 1: Figure S2) [36]. LEfSe analysis identified increased relative abundance of *Prevotella* and *Streptococcus* spp. and reduced relative abundance of *Lactobacillus* spp. as being discriminatory for sporadic euploid miscarriage compared to viable term pregnancies (Fig. 5e).

### Relationship between vaginal microbiota and cytokines concentrations across the entire cohort

**Fig. 6:**
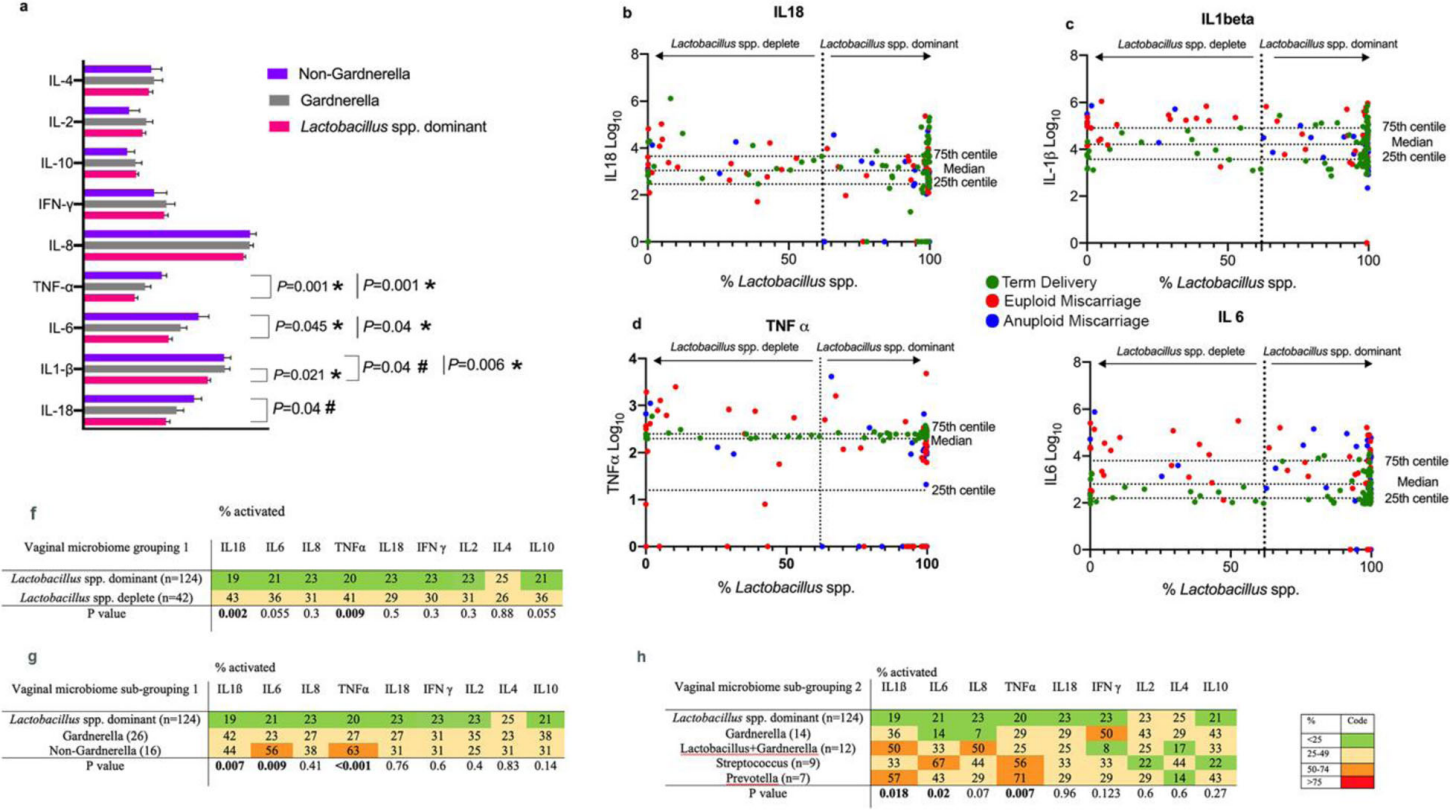
Cytokine expression according to vaginal microbiome grouping 1 and subgroupings 1 and 2. Non-Gardnerella, *Lactobacillus spp.* depleted samples (16/166) had significantly increased vaginal levels of inflammatory cytokines TNF-α, IL-6, IL-1 β and IL-18 when compared to *Lactobacillus spp.* dominant samples (124/166). Gardnerella, *Lactobacillus spp.* depleted samples (26/166) had significantly increased IL-1 β when compared to *Lactobacillus spp.* dominant (**a**, *P values, Kruskal-Wallis test with post hoc Bonferroni correction and ^#^*P* values, Mann-Whitney *U* test for non-Gardnerella versus *Lactobacillus spp.* dominant patients). Scatter plots representing the percentage of *Lactobacillus* spp. against the concentration of IL-18 (**b**), IL-1 β (**c**), TNF-α (**d**), IL-6 (**e**) and coloured according to different clinical outcomes with the 25th, median, and 75th centile of cytokine levels indicated. *Lactobacillus spp.* depleted samples had significantly increased levels of IL-1β and TNF-α compared to the *Lactobacillus spp.* dominated group (**f**). Increased inflammatory activation in the *Lactobacillus spp.* depleted samples could be largely attributed to non-Gardnerella dominated samples (**g**) and more specifically, high relative abundances of *Streptococcus* and *Prevotella* spp. (**h**). Data represented as a clustered bar chart with mean ± standard error of the mean for each cytokine by vaginal microbiome sub-grouping 1, *n* = 166 (**a**). The individual tables (**f**–**h**) illustrate the percentage of activated cytokines in each group. Activation was defined by cytokines expressed in the upper quartile. *P* values obtained by comparing activated versus non-activated cytokines using two-tailed Fisher’s exact test (**f** and **g**) and chi-squared test (**h**)

To explore the relationship between the vaginal microbiota and cervicovaginal inflammatory markers, we compared the levels of nine cytokines between women with *Lactobacillus* spp. depleted or *Lactobacillus* spp. dominated VMG irrespective of pregnancy outcome. The *Lactobacillus* spp. depleted non-Gardnerella VMG sub-group had significantly higher concentrations of tumour necrosis factor (TNF)-α, interleukin (IL)-6, IL-1β and IL-18 compared to *Lactobacillus* spp. dominated VMG (*P* = 0.001,0.045,0.04,0.04 respectively, Fig. 6a). The individual data points for these four cytokines (TNF-α, IL-6, IL-1β and IL-18), their positions within the concentration range quartiles and their relationship to the proportion of *Lactobacillus* spp. relative abundance in the different pregnancy outcome groups is shown in Fig. 6b–e. High cytokine levels for TNF-α and IL-1β (defined as concentrations in the upper quartile) were more frequently observed in the *Lactobacillus* spp. depleted group (*P* = 0.009 and 0.002 respectively, two-tailed Fisher’s exact test, Fig. 6f), particularly non-Gardnerella dominated samples, which also had elevated IL6 levels (*P* = 0.009, two-tailed Fisher’s exact test, Fig. 6g). Further subdivision of the VMG groups showed that increased levels of TNF-α and IL-1β were largely associated with *Prevotella* spp. dominance (*P* = 0.007 and 0.018 respectively, chi-squared test) whereas IL6 was linked to *Streptococcus* dominance (*P* = 0.02 chi-squared test, Fig. 6 h).

### Relationship between vaginal microbiota, cytokine concentrations and pregnancy outcome

**Fig. 7:**
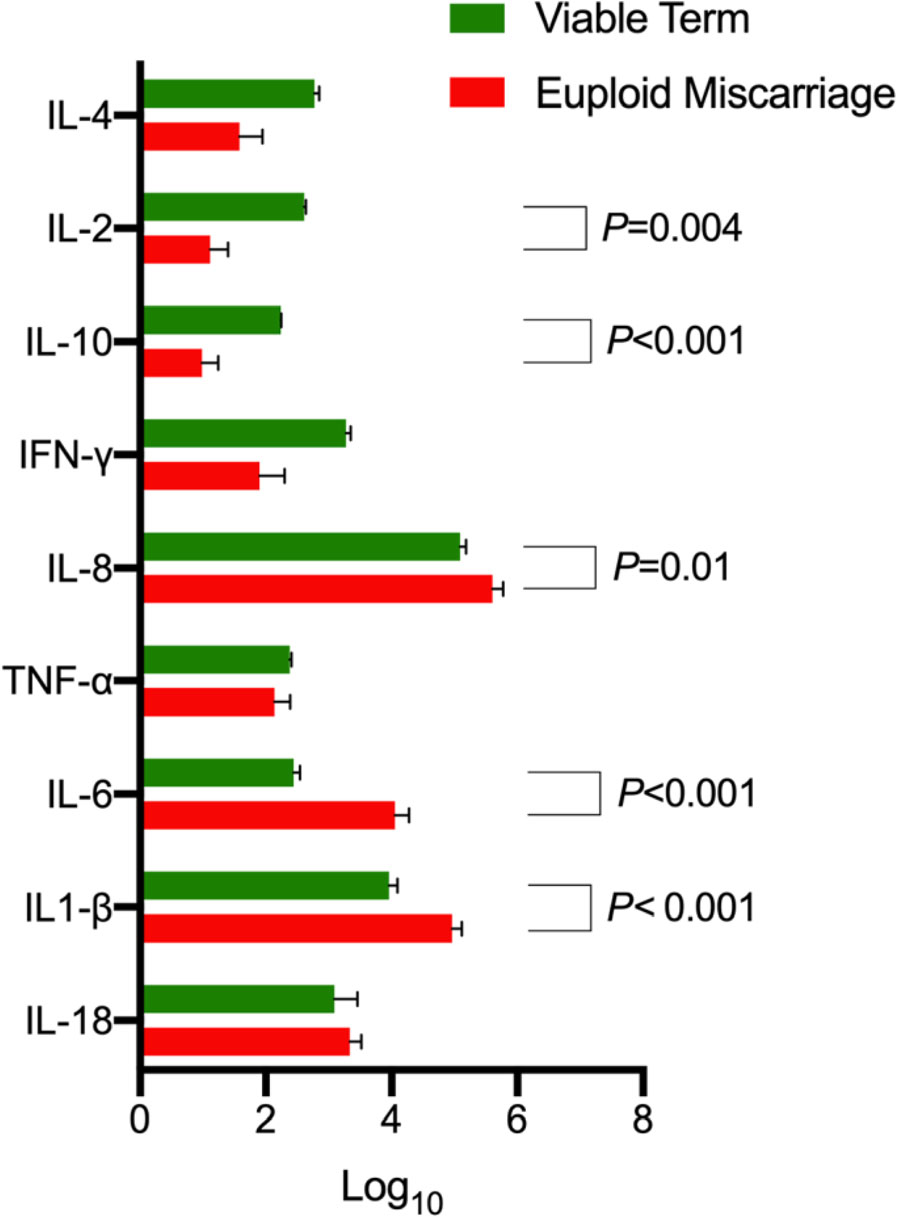
Vaginal cytokine levels according to pregnancy outcome in patients with *Lactobacillus spp.* depleted vaginal microbial composition (*n* = 37, euploid *n* = 20 and viable term *n* = 17). In women with *Lactobacillus spp.* depleted vaginal microbial compositions, viable pregnancy was associated with significantly higher levels of anti-inflammatory cytokines (IL-2 *P* = 0.004, IL-10 *P* < 0.001, two tailed Mann-Whitney *U* test) and lower levels of pro-inflammatory cytokines (IL-6, *P* < 0.001, IL-1ß *P* < 0.001, and IL-8 *P* = 0.01, two tailed Mann Whitney *U* test) compared to euploid miscarriage. Data represented as clustered bar chart with mean ± standard error of the mean for each cytokine according to clinical outcome

The proportion of *Lactobacillus* spp. depleted vaginal microbiota in the aneuploid miscarriage, euploid miscarriage and viable pregnancy groups was, 13%, 37% and 23% respectively. Since nearly a quarter of the pregnancies with a viable outcome nevertheless had a *Lactobacillus* spp. depleted vaginal microbiota, we next compared cytokine concentrations between euploid miscarriage and viable term pregnancies in only women with *Lactobacillus* spp. depletion. IL-1β, IL-6 and IL-8 were significantly lower (*P* < 0.001, *P* < 0.001 and *P* = 0.01, respectively, two tailed Mann-Whitney *U* test) and IL-2 and IL-10 were significantly higher (*P* = 0.004 and *P* < 0.001 respectively, two tailed Mann-Whitney *U* test) in viable pregnancies (Fig. 7).

## Discussion

We confirmed our primary hypothesis that euploid miscarriage is significantly more frequently associated with *Lactobacillus* spp. depleted vaginal microbial communities compared to aneuploid miscarriage. This association was reflected in increased richness and diversity in euploid miscarriage. A *Lactobacillus spp.* depleted vaginal microbiota characterized by Streptococci was the most significant vaginal microbiota risk factor for sporadic euploid miscarriage and the principal driver of proinflammatory mediators in these patients. We also found that aneuploid miscarriages and healthy pregnancies had similar vaginal microbial compositions. These data support the notion that aneuploid and euploid miscarriages generally have different causal mechanisms. In general, aneuploid miscarriage is assumed to be due to a genetic intrinsic failure whereas a proportion of sporadic euploid miscarriage are due to host-vaginal microbe interactions.

Of the 200 patients recruited to this study, 119 eventually miscarried and 81 went on to have a viable pregnancy. The relatively high rate of miscarriage in this cohort was because the majority of miscarriage patients (*n* = 92) were recruited at the time of miscarriage diagnosis. Molecular cytogenetics was available for 93 miscarriage patients, and results were reported after recruitment and sample collections, therefore eliminating selection bias. The proportion of aneuploid pregnancies in our miscarriage cohort is comparable with the aneuploidy rate reported in previous studies [37, 38]. In our cohort, there was a significantly higher maternal age in the aneuploidy miscarriage group, which is consistent with the known relationship between maternal age and the incidence of meiotic errors in embryos [39]. There was a significant difference in the gestational age at sampling between the viable pregnancies and miscarriages likely because women experiencing symptoms in a viable pregnancy tend to present at a later gestational age to the clinic. As expected, there was a significantly higher bleeding score in the miscarriage cohort compared to the viable term group [21]. Recurrent miscarriage is thought to be aetiologically different to sporadic miscarriage and recent studies have highlighted the association with abnormal endometrial function, caused by lack of mesenchymal stem cells and heightened cellular senescence during the midluteal implantation window [40–42]. Our data supports the relationship between miscarriage and vaginal microbiota composition principally in sporadic miscarriage. Although the numbers of recurrent miscarriages in our cohort were small (*n* = 26) and the findings require confirmation in larger populations, this does reinforce the concept that different underlying causal mechanisms drive recurrent miscarriage.

The vaginal microbiota is an important regulator of innate immune response in the reproductive tract [18].

A significant body of evidence has linked inflammation within the cervicovaginal niche to adverse pregnancy outcomes, particularly in relation to second trimester pregnancy loss and preterm birth [43–47]. Consistent with previous findings in pregnant [18] and non-pregnant women [48, 49], we found that proinflammatory cytokines IL-1β, IL-6 and TNF-α were elevated in women with *Lactobacillus* spp. depleted VMGs. These findings support our primary hypothesis and reinforce the view that *Lactobacillus* spp. depleted vaginal microbiota correlate with local inflammatory activation which during pregnancy, can associate with adverse pregnancy outcome [18]. However, a *Lactobacillus* spp. depleted VMG was compatible with delivery at term in nearly a quarter of cases. Our exploration of local immune mediators revealed that women with a *Lactobacillus* spp. depleted VMG who delivered at term had comparably lower levels of proinflammatory cytokines in early pregnancy. Further, although no difference in the levels of anti-inflammatory cytokines IL-2 and IL-10 were observed between *Lactobacillus* spp. depleted and *Lactobacillus* spp. dominated VMGs across the whole cohort, there were significantly increased levels of these cytokines in *Lactobacillus* spp. depleted samples from viable term pregnancies compared to euploid miscarriage. Collectively, these observations implicate both adverse vaginal microbiota composition and specific local host immune responses in early pregnancy with subsequent risk of miscarriage.

In this study, *Gardnerella vaginalis* was observed as being an important feature in high risk *Lactobacillus* spp. deplete compositions. Recent work has indicated the likely existence of difference *Gardnerella vaginalis* clades with potentially varying degrees of pathogenicity [50]. However, the metaxonomics approach used in our study was not capable of differentiating these. Future investigations may shed light on whether particular *Gardnerella vaginalis* clades are responsible for the observed relationship with adverse outcomes.

Furthermore, in our cohorts increased production of proinflammatory cytokines (IL-6, IL-1β, IL-18 and TNF-α) within the *Lactobacillus* spp. depleted VMGs was associated with dominance by *Prevotella* and *Streptococcus* species in women with euploid miscarriages. These species had low co-occurrence with *L. crispatus*, which has been shown to associate with vaginal bacterial stability and low levels of innate immune activation during pregnancy [18, 51]. Thus, whilst the underlying microbial structure may be the primary driver, it is the interplay between the vaginal microbiota and host immune response that determines the amplitude of the inflammatory response and the likelihood of miscarriage.

There are a number of potential mechanisms by which vaginal microbiota and host immune response could be causally linked to miscarriage. Suboptimal vaginal microbiota characterised by *Lactobacillus* spp. depletion and high bacterial diversity are associated with local inflammatory activation and damage to the cervical epithelial barrier [52] that can promote bacterial translocation [53]. Although studies of the endometrial microbiome are confounded by the difficulties of contamination and low biomass, the emerging evidence is that the lower uterine microbiome is distinct but maybe contributed to by the vaginal microbiota [54, 55]. In this study, microbiota and cytokine measurements were limited to CVF, thus it is not possible to determine if our observations are reflective of microbiota-host interactions in the endometrial mucosa. Logistical and ethical considerations make direct sampling of the early pregnancy uterine environment difficult. However, the embryological origin of Mullerian duct fusion means that the upper one third of the vagina shares similarities with the endometrial epithelium and therefore a similar proinflammatory response to pathobionts such as *Prevotella and Streptococcus* species would be expected [56, 57].

Lactobacilli confer protection in the vagina by promoting antimicrobial defense without initiating innate immune mediated inflammation [14]. Therefore it is possible in cases of *Lactobacillus* spp. depletion a proinflammatory environment can alter successful implantation, which is a process of tissue injury and repair that is regulated by immune mediators to allow the trophoblast to breach the decidual lining and invade the maternal decidua [5, 58]. Previous studies have also shown that altered levels of cytokines at the maternal fetal interface can trigger early pregnancy complications [59–61].

It is therefore plausible that an inflammatory response triggered by the vaginal microbiome could directly or indirectly contribute to dysregulation of the maternal decidua, promoting breakdown of the nascent maternal-fetal interface in early gestation [9].

## Conclusions

In conclusion, we demonstrate that vaginal microbiota depleted of *Lactobacillus* spp. combined with a heightened local inflammatory response, predispose pregnant women to euploid miscarriage. Although this may be a reflection of intrinsic maternal immune response, it appears that the cytokine response is largely driven by the bacterial taxa present in the vagina, which presents an opportunity for specific, directed intervention prior to conception or in early pregnancy. First trimester miscarriage occurs in 20% of pregnancies and is a major cause of physical and psychological pathology worldwide [62]. Whilst aneuploid miscarriage can be explained in terms of an intrinsic developmental defect, there is currently little evidence to explain the causative mechanisms underlying sporadic euploid miscarriage. There are currently no treatments to prevent this important clinical condition. The data presented here suggests that there is a group of women who would benefit from antibiotic or pre- or probiotic treatment to reduce the risk of miscarriage. Whilst further studies are needed to validate these findings and to understand what specifically triggers an inflammatory cascade in euploid miscarriage, an important next step will be to explore which interventional regimes might change the vaginal microbiota and positively influence pregnancy outcome.

## A.Additional files

**Additional file 1: Figure S1** Heat map of relative abundance data for the top 50 most prevalent vaginal bacterial species and relationship with different clinical outcomes (*n*=167). **Figure S2** Clinical outcomes according to vaginal microbial composition in sporadic miscarriage. Patients with recurrent miscarriage (*n*=13) were excluded using the stricter criteria ( ≥3 miscarriages with no live birth). **Figure S3** A key of code names for the co-occurrence network analysis of vaginal bacterial taxa in Fig. 3.

## Data Availability

The datasets used and/or analysed during the current study are available from the corresponding author on reasonable request.

## Abbreviations

PPROM: Preterm prelabour rupture of fetal membranes
CVF: Cervicovaginal fluid
LMP: Last menstrual period
CRL: Crown-rump length
QF-PCR: Quantitative fluorescent polymerase chain reaction
BACs: Bacterial artificial chromosomes
OUT: Operational taxonomic units
VMG: Vaginal microbiome groups
BMI: Body mass index
DNA: Deoxyribonucleic acid
LDA: Latent discriminatory analysis
LEfSe: Linear discriminant analysis with effect size
