# Supplementary material for: Chromosomally normal miscarriage is associated with vaginal dysbiosis and local inflammation
Source: BMC Med. 2022 Jan 28;20:38. doi: 10.1186/s12916-021-02227-7 (PMC8796436; doi:10.1186/s12916-021-02227-7)
Supplement: Supplementary file 1 — Additional file 1: Figure S1 Heat map of relative abundance data for the top 50 most prevalent vaginal bacterial species and relationship with different clinical outcomes (n=167). Figure S2 Clinical outcomes according to vaginal microbial composition in sporadic miscarriage. Patients with recurrent miscarriage (n=13) were excluded using the stricter criteria ( ≥3 miscarriages with no live birth). Figure S3 A key of code names for the co-occurrence network analysis of vaginal bacterial taxa in Fig. 3. [file 12916_2021_2227_MOESM1_ESM.docx]

**
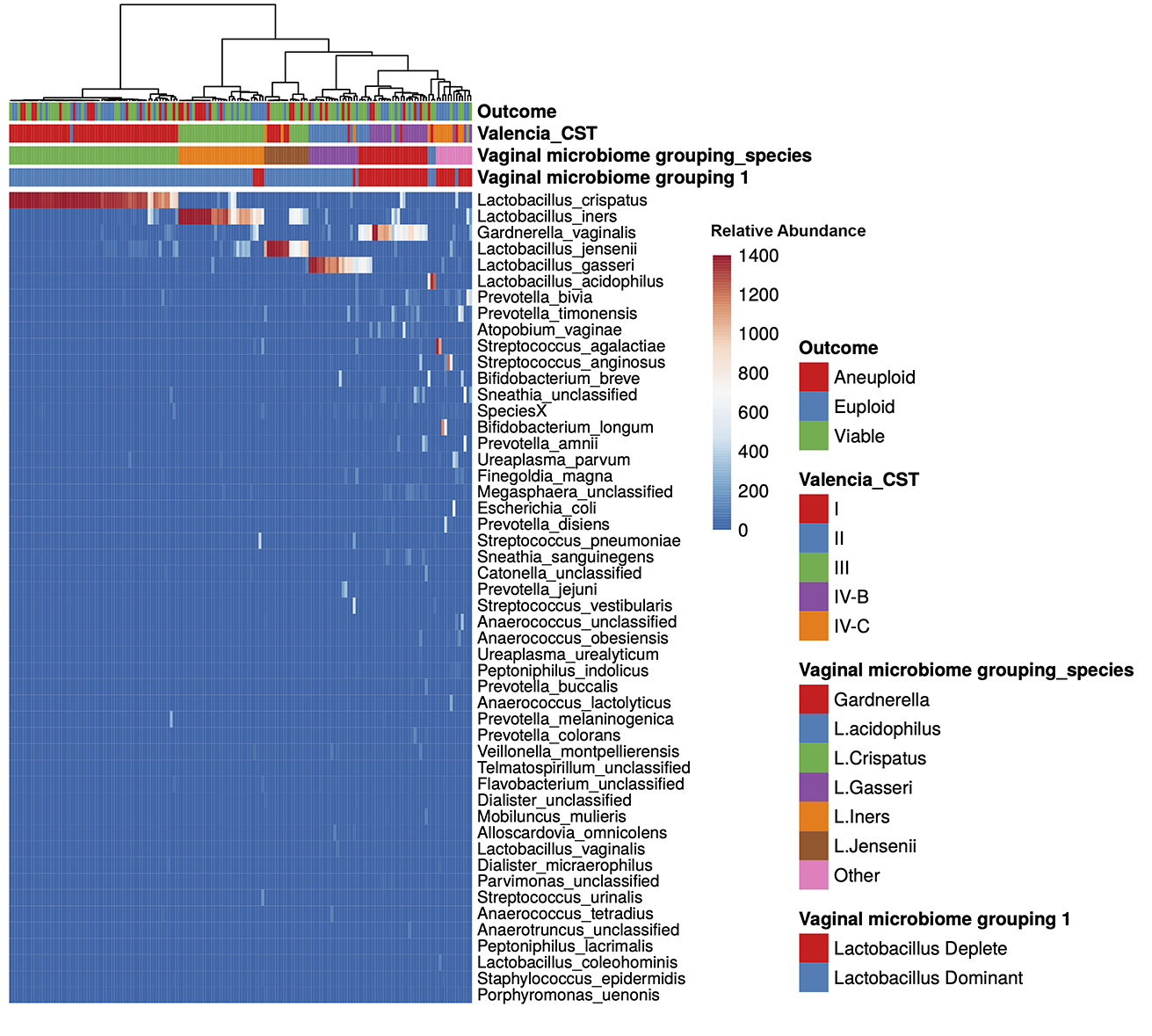
**

**Additional file 1:Figure S1. Heat map of relative abundance data for the top 50 most prevalent vaginal bacterial species and relationship with different clinical outcomes (n=167).**

Mixed universal primers 28F-YM GAGTTTGATYMTGGCTCAG, 28F-Borrellia GAGTTTGATCCTGGCTTAG, 28F-Chloroflex GAATTTGATCTTGGTTCAG and 28F-Bifdo GGGTTCGATTCTGGCTCAG at a ratio of 4:1:1:1 with 388R reverse primers were used to amplify the V1-V2 region of 16S rRNA followed by sequencing using the Illumina MiSeq platform. The data was processed using the MiSeq SOP Pipeline of the Mothur package and classification used RDP. Ward hierarchical clustering separated patient samples in clusters that were *Lactobacillus crispatus* (37%), *Lactobacillus* *iners* (19%), *Lactobacillus gasseri* (11%), *Lactobacillus* *jensenii* (10%), *Lactobacillus* *acidophilus* (2%), *Gardnerella* *vaginalis* (15%), and a highly diverse group (8%).

**
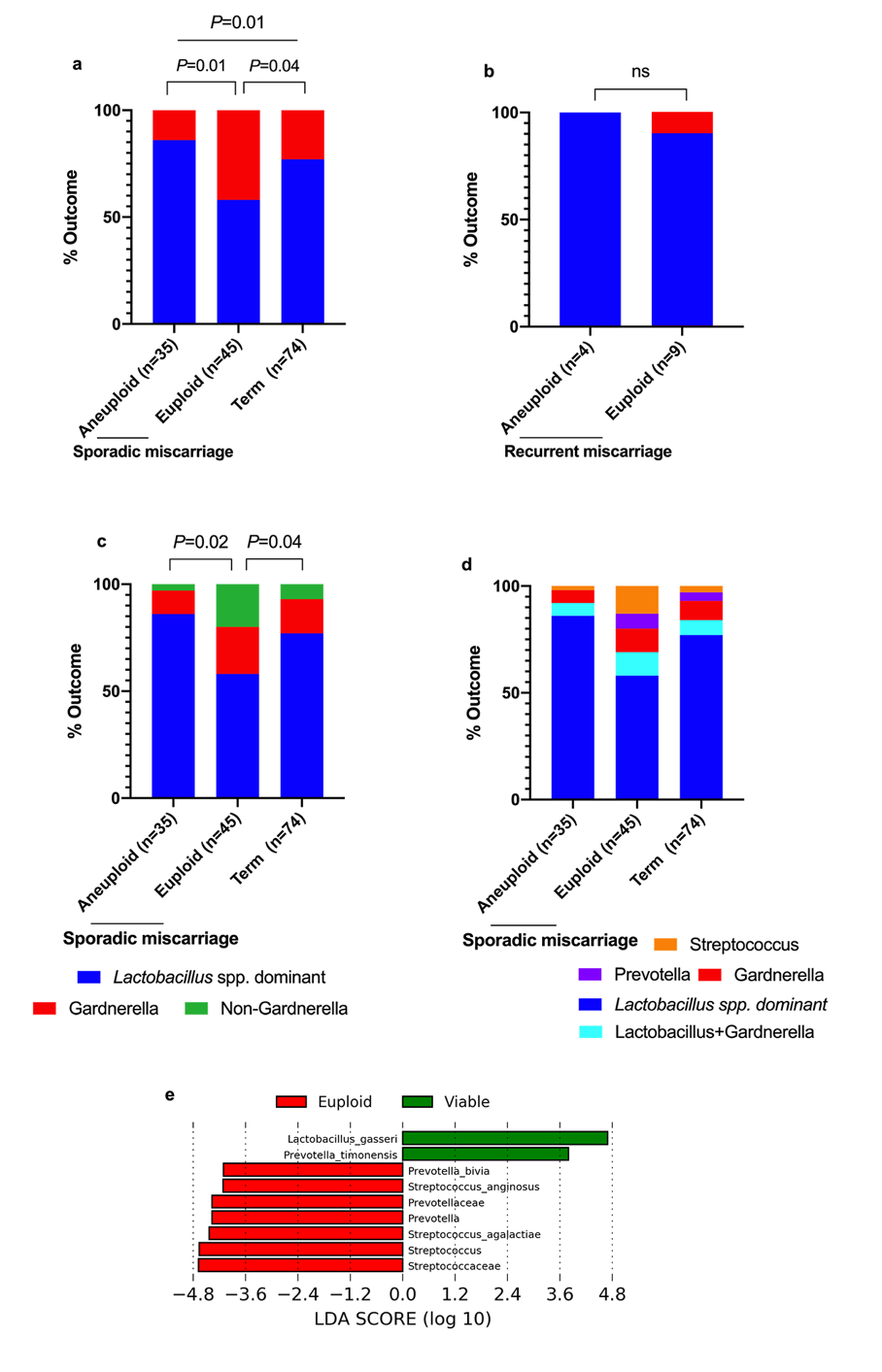
**

**Additional file 1: Figure S2. Clinical outcomes according to vaginal microbial composition in sporadic miscarriage. Patients with recurrent miscarriage (n=13) were excluded using the stricter criteria ( ≥3 miscarriages with no live birth).** Increased *Lactobacillus spp.* depleted vaginal microbial communities were observed in sporadic euploid miscarriages compared to sporadic aneuploid miscarriages (*P*=0.01 two-tailed Fisher’s exact test) and viable term pregnancies (*P*=0.04 two-tailed Fisher’s exact test, **a**). No significant difference was seen between euploid and aneuploid miscarriages in the recurrent miscarriage group (**b**). A significantly increased prevalence of Non-Gardnerella vaginal bacterial communities was seen in euploid miscarriages compared to aneuploid miscarriage, (*P*=0.02 two-tailed Fisher’s exact test) and viable pregnancy (*P*=0.04 two-tailed Fisher’s exact test, **c**). Data represented as percentages in **a**, **b**, **c** and **d.** Differentially abundant taxa identified by LDA in euploid miscarriage compared to viable pregnancy (**e**).

**a**

| **Genus** | **Code** |
| --- | --- |
| Timonensis | Tim |
| Bifidobacterium | Bfd |
| Lactobacillus | Lact |
| GeneraX | Gen |
| Atopobium | Atp |
| Gardnerella | Gard |
| Alloscardovia | Als |
| Sneathia | Sne |
| Prevotella | Prev |
| Finegoldia | Fng |
| Parvimonas | Parv |
| Anaerotruncus | Anaero |
| Megasphaera | Meg |
| Dialister | Dls |
| Anaerococcus | Anaer |
| Streptococcus | Strep |
| Peptoniphilus | Ppt |
| Porphyromonas | Prp |
| Mobiluncus | Mob |
| Flavobacterium | Flv |
| Catonella | Cat |
| Staphylococcus | Staph |
| Ureaplasma | Ure |
| Veillonella | Vel |
| Escherichia/Shigella | Shg |

**b**

| **Species** | **Code** |
| --- | --- |
| Alloscardovia_omnicolens | Al_ |
| Anaerococcus_lactolyticus | AN_l |
| Anaerococcus_obesiensis | An_b |
| Anaerococcus_tetradius | An_t |
| Anaerococcus_unclassified | An_l |
| Anaerotruncus_unclassified | Atr_ |
| Atopobium_vaginae | At_v |
| Bifidobacterium_breve | Bf_b |
| Bifidobacterium_longum | Bf_l |
| Catonella_unclassified | Ct_ |
| Dialister_micraerophilus | Dl_m |
| Dialister_unclassified | Dl_n |
| Escherichia_coli | E_c |
| Finegoldia_magna | Fn_ |
| Flavobacterium_unclassified | Fl_ |
| Gardnerella_vaginalis | Gr_ |
| Lactobacillus_acidophilus | L_cd |
| Lactobacillus_coleohominis | L_cl |
| Lactobacillus_crispatus | L_cr |
| Lactobacillus_gasseri | L_g |
| Lactobacillus_iners | L_n |
| Lactobacillus_psittaci | L_j |
| Lactobacillus_vaginalis | L_v |
| Megasphaera_unclassified | Mg_ |
| Mobiluncus_mulieris | Mb_ |
| Parvimonas_unclassified | Prv_n |
| Peptoniphilus_indolicus | Pp_n |
| Peptoniphilus_lacrimalis | Pp_l |
| Porphyromonas_uenonis | Prp_ |
| Prevotella_amnii | Prv_am |
| Prevotella_bivia | Prv_bv |
| Prevotella_buccalis | Prv_bu |
| Prevotella_colorans | Prv_c |
| Prevotella_disiens | Prv_d |
| Prevotella_jejuni | Prv_j |
| Prevotella_melaninogenica | Prv_m |
| Prevotella_timonensis | Prv_t |
| Sneathia_sanguinegens | Sn_s |
| Sneathia_unclassified | Sn_n |
| SpeciesX | Sp_ |
| Staphylococcus_epidermidis | Stp |
| Streptococcus_agalactiae | Stp_g |
| Streptococcus_anginosus | Stp_n |
| Streptococcus_pneumoniae | Stp_p |
| Streptococcus_urinalis | Stp_r |
| Streptococcus_vestibularis | Stp_v |
| Telmatospirillum_unclassified | Tl_ |
| Ureaplasma_parvum | Ur_p |
| Ureaplasma_urealyticum | Ur_r |
| Veillonella_montpellierensis | V_m |

**Additional file 1: Figure S3**

A key of code names for the co-occurrence network analysis of vaginal bacterial taxa in Figure 3.
